# Virus-like particle encapsulation of functional proteins: advances and applications

**DOI:** 10.7150/thno.103127

**Published:** 2024-11-04

**Authors:** Xianxun Sun, Yindong Lian, Tao Tian, Zongqiang Cui

**Affiliations:** 1 School of Life Sciences, Jianghan University, Wuhan 430056, China.; 2 State Key Laboratory of Virology, Wuhan Institute of Virology, Center for Biosafety Mega-Science, Chinese Academy of Sciences, Wuhan 430071, China.

**Keywords:** virus-like particle, imaging, therapeutic enzyme, vaccine, immunotherapy, nanoreactor, biosensor

## Abstract

Proteins face several challenges in biomedicine, including issues with antibody production, degradation by proteases, rapid clearance by the kidneys, and short half-lives. To address these problems, various nano delivery systems have been developed, with virus-like particles (VLPs) emerging as a leading solution. VLPs, which are self-assembled protein complexes, offer effective encapsulation and transport of proteins. They provide enhanced stability, extended circulation time, preserved biological activity, improved targeting for therapies or imaging, and reduced side effects due to minimized systemic exposure. This review explores various methods for encapsulating proteins within VLPs. It assesses the benefits and limitations of each method and their applications in imaging, therapeutic enzyme delivery, vaccines, immunotherapy, nanoreactors, and biosensors. Future advancements in VLPs will depend on improving packaging methods, controlling protein loading, optimizing assembly techniques, and enhancing capsid design. The review also discusses current challenges and proposes solutions to advance the use of VLPs in various applications.

## 1. Introduction

In biomedicine and biochemistry, the use of various types of proteins is becoming increasingly common. However, these proteins encounter several challenges 1, 2. For example, therapeutic proteins can suffer from issues such as antibody generation, protease degradation, rapid kidney clearance, short circulation half-life, and lack of specificity 3, 4. Fluorescent proteins (FPs) used in imaging face problems like low concentration and susceptibility to photobleaching. Catalytic enzymes often become denatured when exposed to extreme pH levels or high temperatures 5, 6. Additionally, proteins used as antigens may not always have sufficient immunogenicity 7. To address these issues, researchers have developed a range of drug delivery nanocarriers designed to improve protein stability and delivery 8-11. These include liposomes, micelles, dendritic polymers, polymer particles, carbon nanotubes, and virus-like particles (VLPs). Among these, VLPs have garnered significant attention due to their unique properties and potential advantages 12, 13.

**Figure 1:**
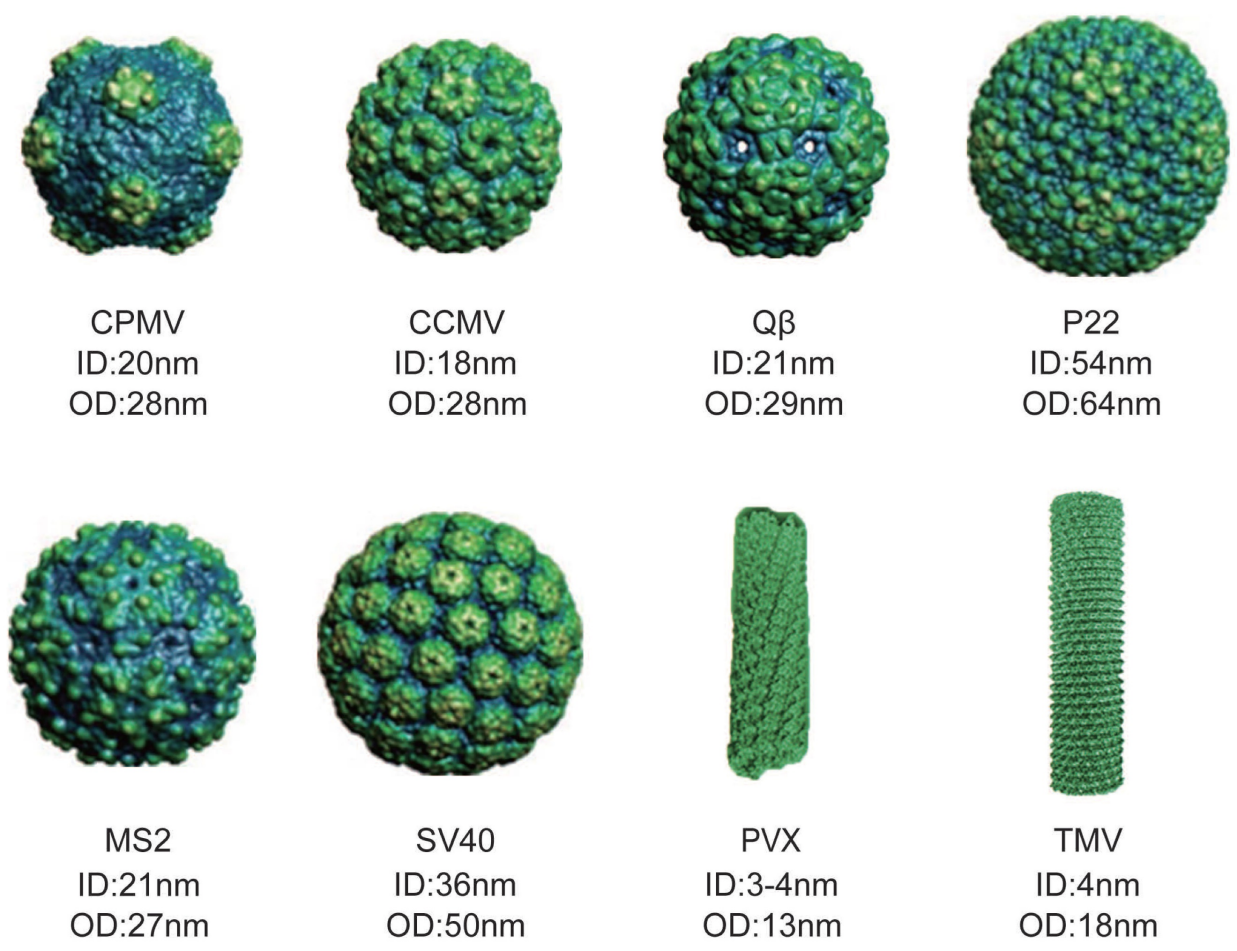
Various sizes and shapes of VLPs deriving from different viruses. The figure illustrates model of cowpea mosaic virus (CPMV), cowpea chlorotic mottle virus (CCMV), Qβ, P22, MS2, simian virus 40 (SV40), potato virus X (PVX) and tobacco mosaic virus (TMV). (Reproduced with permission from Ref. 25 and Ref. 26. Copyright 2014 Wiley-VCH Verlag; Copyright 2019 Multidisciplinary Digital Publishing Institute).

VLPs are self-assembled protein complexes that mimic the shape and size of real viruses 14-16. They are typically either icosahedral or rod-shaped, and they maintain a precise structure and uniform size 17-21. The shape and size of VLPs can vary depending on their source, with each particle having different inner diameter (ID) and outer diameter (OD) (Figure 1) 22. VLPs offer several advantages as natural delivery nanocarriers. They have a uniform morphology, are biocompatible, water-soluble, and easily customizable. Their hollow structure, whether icosahedral or filamentous, makes them suitable for delivering proteins in high concentrations, either within their internal cavity or on their exterior surface. The active groups on their surface can be modified to attach specific ligands for targeted therapy or imaging, minimizing systemic circulation 23. Furthermore, VLP surfaces can be coated with non-immunogenic polymers or proteins to evade the immune system. Their porous nature allows for the entry of substrates and exit of products, while providing a contained environment for simultaneous reactions 24. These features make VLPs highly effective as vehicles for protein delivery.

Viruses have developed various strategies to package their genomes, minor capsid proteins (CPs), and nonstructural proteins, and these strategies can also be applied to encapsulate external proteins using VLPs. The properties and assembly mechanisms of VLPs influence the methods available for protein encapsulation. This review begins by summarizing the different techniques used for encapsulating proteins within VLPs. These include random encapsulation, charge-mediated encapsulation, coiled-coil-mediated encapsulation, DNA or RNA aptamer-mediated encapsulation, SpyTag/SpyCatcher-mediated encapsulation, scaffolding protein-mediated encapsulation, covalent targeted encapsulation, and SrtA-mediated encapsulation. Each method is evaluated for its advantages and limitations. The review also explores the diverse applications of VLPs in protein encapsulation, highlighting their roles in imaging, therapeutic enzyme delivery, vaccines and immunotherapy, nanoreactors, and biosensors. With ongoing advancements in encapsulation techniques, the scope of VLP applications continues to expand 27-30. However, there is a gap in comprehensive reviews covering these methods and applications, underscoring the need for an updated overview of recent developments in this field.

## 2. Structural characteristics of VLPs

VLPs have distinctive structural features that make them highly effective as protein nanocarriers 31-33. Their unique composition and structural characteristics stem from their viral origins, where the amino acid sequences of the CPs are determined by the viral genetic material. By employing genetic engineering techniques, these sequences can be precisely altered, enabling the incorporation of functional molecules at specific locations on the CPs 34, 35. Genetic modifications not only influence the shape and stability of VLPs but also their production, which is critical for their role as protein nanocarriers. Due to their symmetrical structure, any genetic changes are uniformly distributed across the VLP, ensuring consistent modification throughout the particle. Unlike synthetic nanocapsules or lipid-based carriers, the genetic manipulation of VLP subunits allows for the precise placement of cysteine or non-natural amino acids, creating specific sites for chemical conjugation. This facilitates the encapsulation or display of proteins and other molecules. Additionally, VLPs can be engineered to introduce large numbers of amino acids, such as lysine, which can be chemically modified to attach multiple ligands. These capabilities enhance the versatility of VLPs as nanocarriers.

VLPs feature three key interfaces: the outer surface, the inner surface, and the inter-unit interface, each providing different functional possibilities. The outer surface can be modified to display multiple ligands, including cell-targeting or cell-penetrating peptides, tailored for specific cell types or tissues, which improves cellular uptake. The inner surface offers a platform for embedding proteins or enzymes within the VLP cavity. Furthermore, adjusting interactions between subunits can alter VLP stability and control pore permeability. VLPs with different pore sizes can accommodate small molecules, making them useful as nanoreactors or biosensors. These structural features enhance their utility for protein delivery and controlled release applications.

## 3. Methods for VLPs encapsulating proteins

Successful encapsulation of proteins in VLPs depends on various factors, including the size of the protein cargo, surface charge, electrostatic interactions, and hydrophobic or hydrophilic properties 36. Different techniques have been developed for this purpose, ranging from leveraging natural biomolecular interactions to using specialized affinity tags on either the VLP CPs or the target proteins 37.

**Figure 2:**
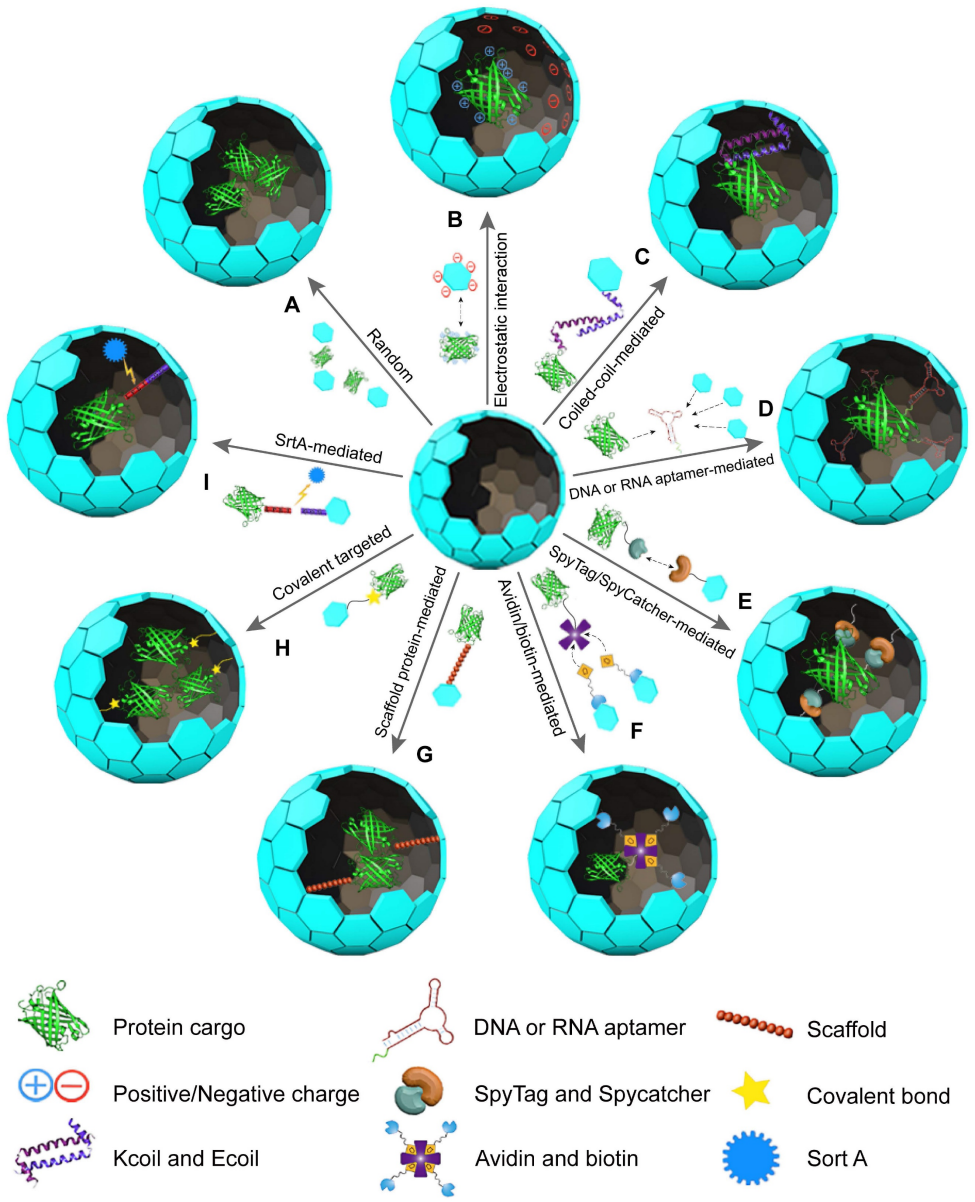
Illustration of protein encapsulation methods into VLPs: (A) Random encapsulation, (B) Charge-mediated encapsulation, (C) Coiled-coil encapsulation, (D) DNA or RNA aptamer-mediated encapsulation, (E) SpyTag/SpyCatcher-mediated encapsulation, (F) Avidin/biotin-mediated encapsulation, (G) SP-mediated encapsulation, (H) Covalent targeted encapsulation, (I) SrtA-mediated encapsulation.

This section explores the use of VLPs as vehicles for protein delivery, focusing on methods for either encapsulating proteins inside the VLP cavity or displaying them on the VLP surface. Nine widely used methods for protein encapsulation in VLPs include (Figure 2): (1) Random encapsulation: proteins are incorporated into VLPs without specific targeting. (2) Electrostatic interaction-mediated encapsulation: proteins are captured within VLPs through electrostatic forces. (3) Coiled-coil-mediated encapsulation: proteins are enclosed in VLPs using coiled-coil interactions. (4) DNA or RNA tag-mediated encapsulation: specific nucleic acid tags facilitate the encapsulation of proteins. (5) SpyTag/SpyCatcher-mediated encapsulation: this method uses SpyTag and SpyCatcher proteins to secure proteins within VLPs. (6) Avidin/Biotin-mediated encapsulation: biotinylated proteins are captured by VLPs through avidin-biotin interactions. (7) Scaffolding protein (SPs)-mediated encapsulation: SPs are used to mediate protein encapsulation. (8) Covalent targeted encapsulation: proteins are covalently bonded to the VLPs for stable encapsulation. (9) Ligase-mediated encapsulation: protein incorporation is achieved through ligase-mediated reactions. Each method has distinct advantages and limitations, which are discussed in detail below.

### 3.1 Random encapsulation

VLPs can be disassembled and reassembled by altering buffer conditions, a feature that has been effectively used to encapsulate guest proteins 38. This process relies on a simple self-assembly mechanism, where VLPs can encapsulate proteins within their cavities when specific conditions are met (Figure 2A) 39-41. In random encapsulation, proteins are mixed with dissociated VLPs in a solution. Adjusting the conditions to promote self-assembly leads to the proteins being randomly encapsulated 42, 43. For instance, CCMV VLPs can reversibly assemble and disassemble with changes in pH from 5.0 to 7.5, allowing them to encapsulate enzymes like horseradish peroxidase (HRP). This property is particularly useful for studying enzyme kinetics 44. Similarly, hepatitis B virus core particles (HBVc) have been used to encapsulate green fluorescent protein (GFP) 42. The ability to control VLP assembly through chemical conditions provides a notable advantage in managing the average number of proteins encapsulated within each VLP 45. This method avoids the need for complex chemical reactions, relying instead on reversible assembly processes 46. However, random encapsulation tends to have lower efficiency compared to methods involving covalent bonding. It also requires a larger amount of purified proteins, which must be removed after the VLPs reassemble. Additionally, this approach does not guarantee that every VLP will successfully encapsulate the protein, limiting the overall amount of protein cargo that can be loaded 43, 47.

### 3.2 Electrostatic interaction-mediated encapsulation

Electrostatic interaction-mediated encapsulation leverages the complementary charges between VLPs and proteins to facilitate protein packaging (Figure 2B). The positively charged interiors of VLPs are well-suited for encapsulating negatively charged proteins 48, 49. For instance, Qβ VLPs can encapsulate red-shifted FPs through these electrostatic interactions 50. Similarly, bacteriophage Qβ and PP7 VLPs can hold about three small-ultrared fluorescent proteins (smURFP) per particle 51. MS2 VLPs have also been used to encapsulate *E. coli* alkaline phosphatase (PhoA), with enhanced efficiency achieved by adding a negatively charged peptide tag to PhoA 52. This modification allows MS2 VLPs to encapsulate approximately 1.6 PhoA molecules while preserving enzyme activity 52. Enhanced GFP and other proteins, like renilla luciferase, can be similarly encapsulated within VLPs by introducing negatively charged tags or oligopeptides 53, 54.

Another strategy involves using nucleic acids to add negative charges to the protein cargo, facilitating encapsulation 55. The CCMV VLPs, for example, can bind to negatively charged proteins modified with nucleic acids 55. This approach allows for the encapsulation of one glucose oxidase (GOx) or a combination of GOx and gluconokinase per CCMV VLP. RNA, which is essential for CP assembly into VLPs, can be replaced by DNA, enabling the encapsulation of fluorescently labeled streptavidin conjugated to a biotinylated oligonucleotide 56. This method, while providing flexibility and simplicity, generally results in lower loading efficiencies compared to specific interaction methods 37. Nonetheless, it allows for adjustments in loading density by altering the adapter length or ionic strength.

### 3.3 Coiled-coil-mediated encapsulation

The coiled-coil-mediated encapsulation method utilizes the complementary nature of coiled-coil structures to package proteins within VLPs. Coiled-coils are formed by repeating seven-amino acid sequences that create complementary heterodimers known as E-coil and K-coil 57, 58. These motifs, characterized by their repetitive hydrophobic residues, drive the formation of stable coiled-coil structures 59, 60. In this method, the K-coil is introduced to the interior-facing N-terminus of the CCMV CP, while the E-coil is attached to the C-terminus of the target protein, such as EGFP. This setup has enabled the encapsulation of up to 15 EGFP molecules within CCMV VLPs, allowing for greater control over loading density compared to non-specific packaging methods (Figure 2C) 46. Further applications of this approach include the encapsulation of HRP by fusing the E-coil to HRP and the K-coil to the CCMV CP 61. This encapsulation facilitates single-molecule studies of the enzyme. Similarly, Lipase B from Pseudozyma antarctica has been successfully encapsulated using this technique, demonstrating an increased reaction rate compared to free enzymes 62.

The efficiency of enzyme encapsulation can be predicted based on the ratio between the capsid and the enzyme during the coiled-coil-mediated process 63. This method supports single-enzyme catalytic studies and allows for the encapsulation of multiple enzymes with controlled efficiency. While coiled-coil-mediated encapsulation offers a simple and efficient way to achieve high protein loading densities, it is important to consider that genetic modifications to the E-coil or K-coil sequences might affect the functionality of the target protein or the assembly of the VLPs.

### 3.4 DNA or RNA aptamer-mediated encapsulation

DNA and RNA aptamers have been effectively utilized for the encapsulation of proteins in VLPs 64. These aptamers, capable of recognizing specific sequences or structures, are essential for guiding the assembly of capsids. They act as packaging signals, triggering the formation of VLPs and the incorporation of protein cargo (Figure 2D) 50, 65. For instance, the assembly of MS2 VLPs is facilitated by a 19-nucleotide RNA stem-loop. When this RNA stem-loop is attached to a glycoprotein toxin such as ricin toxin A-chain, MS2 VLPs can encapsulate and deliver the toxin into mammalian cells efficiently 65. Similarly, Qβ VLPs rely on an RNA hairpin structure that interacts with the CP interior 66. By using a bifunctional RNA molecule that includes an α-Rev RNA aptamer and a Qβ genome packaging hairpin, Qβ VLPs can encapsulate proteins tagged with an N-terminal Rev peptide. This method has enabled the encapsulation of up to 18 Rev-tagged dipeptidase E enzymes 66. Moreover, this strategy has been applied to encapsulate FPs into Qβ VLPs, with each VLP containing up to 15 proteins or 5-9 copies of near-infrared FPs (NIR-FPs) 67. In this approach, the VLPs form spontaneously when the CP and positively charged Rev peptide-tagged proteins are co-expressed in Escherichia coli cells 68. The use of aptamers simplifies the process by eliminating the need for additional purification and incubation steps found in other methods. Furthermore, encapsulation efficiency can be adjusted by altering expression conditions. In summary, DNA and RNA aptamers offer a straightforward, adaptable, and efficient means of encapsulating proteins in VLPs, demonstrating significant advantages over other encapsulation techniques.

### 3.5 SpyTag/SpyCatcher-mediated encapsulation

The SpyTag/SpyCatcher system offers an efficient method for encapsulating proteins within VLPs. In this system, SpyTag, which contains an active aspartic acid, and SpyCatcher, with an active lysine, form rapid covalent bonds when they interact (Figure 2E) 69. This approach has been successfully applied to MS2 VLPs, where SpyTag is inserted into the internal loop of MS2 CPs, and SpyCatcher is attached to two different enzymes 70. Co-expressing the SpyCatcher-modified enzymes with SpyTag CPs allows each MS2 VLP to encapsulate 2 to 4 enzyme molecules 70. Similarly, SpyCatcher has been linked to FPs like EGFP or mCherry, while SpyTag is fused to the C-terminus of the norovirus VP1 protein. The conjugation efficiency of this setup ranges from 50-63% based on densitometric analysis and 77% based on optical quantification 71. Moreover, using the SpyTag/SpyCatcher system, HBV VLPs can encapsulate over 200 luciferase molecules, increasing detection signals by more than 1500-fold and enabling the visible detection of antigens 72. Compared to Sortase-mediated encapsulation, the SpyTag/SpyCatcher system does not require additional ligases for cargo capture, making it a simpler and more practical choice for *in vivo* applications. However, potential steric constraints from SpyCatcher fusions may limit cargo loading density.

### 3.6 Avidin/biotin-mediated encapsulation

The Avidin/biotin system relies on the strong and specific binding between Avidin, a tetrameric protein with a high affinity for biotin, and biotin, a small molecule that can be conjugated to various cargo proteins 73-76. This strong interaction ensures stable loading of proteins onto VLPs, maintaining their stability under different experimental conditions, including varying reagent concentrations, pH levels, and potential protein denaturing agents 77, 78. By attaching Avidin to VLPs and biotin to target proteins, efficient encapsulation or display of these proteins on VLPs can be achieved (Figure 2F). For example, Tobacco mosaic virus (TMV) was modified with bifunctional linkers to create TMVcys/Bio. Enzymes such as GOx and HRP were separately conjugated to streptavidin, and these enzyme-streptavidin complexes were then attached to the TMV VLPs 79. The resulting GOx/HRP-TMV complexes can be used in biosensors for glucose detection, showing up to 45 times higher catalytic activity compared to controls 79. Other studies have similarly utilized the Avidin/biotin interaction for attaching proteins to biotinylated TMV nanotubes, demonstrating its effectiveness in various biosensor applications 80.

The Avidin/biotin method offers several benefits: the strong interaction ensures stable attachment of cargo proteins, which enhances the stability of VLPs during transport and delivery 81. It is versatile, allowing a wide range of proteins to be loaded onto VLPs, making it suitable for therapeutic and vaccine development 82. Additionally, because the interaction is non-covalent, it minimizes the risk of altering or denaturing the cargo proteins, preserving their bioactivity 83. However, there are limitations, such as potential immunogenicity of Avidin, which might provoke immune responses upon repeated use. Optimization of the conjugation process is crucial to maximize loading efficiency while maintaining VLP integrity and functionality. Additionally, scalability and cost-effectiveness for large-scale production remain important considerations for clinical applications.

### 3.7 Scaffold protein-mediated encapsulation

In certain VLP assembly processes, scaffold proteins (SPs) are essential for the polymerization of CPs 84, 85. Typically, the C-terminal residues of SPs are crucial for forming the capsid, while modifications to the N-terminus, such as truncations or fusions with enzymes, do not impair their function (Figure 2G) 86. For example, the P22 VLP, made up of 420 CPs and 100-330 SPs, assembles into an icosahedral structure 87, 88. By attaching alcohol dehydrogenase D (AdhD) to the N-terminus of SPs, AdhD-P22 VLPs are created with an average of 249 AdhD molecules per particle 89. Similarly, fusing aspartase with SPs results in AsNase-P22 VLPs, which, when coated with PEG, provide an improved biobetter for treating acute lymphoblastic leukemia, featuring enhanced stability and reduced immunogenicity 90. Additionally, P22 VLPs can encapsulate the KivD and AdhA enzymes via SP mediation, forming hierarchical 3D arrays where the encapsulated enzymes maintain catalytic activity and facilitate a coupled reaction to produce isobutanol 91. When green fluorescent protein (GFP) or mCherry is genetically fused to an N-terminally truncated SP and co-expressed with P22 CPs, the resulting VLPs achieve high loading capacities of 281 GFP molecules or 233 mCherry molecules per capsid 85. This high loading ratio results from the 1:1 ratio of guest proteins to SPs 92. While *in vivo* assembly of P22 VLPs simplifies the process by eliminating purification steps, it offers limited control over the stoichiometry and density of the encapsulated proteins 93. To address these issues, adjusting the ratio of cargo-fused SPs to wild-type SPs allows for controlled packaging stoichiometry and density 94.

Research into the impact of SP length on protein packaging has also been conducted. For instance, the murine polyomavirus (MPyV) VLP, composed of 72 pentamers of VP1 and various VP2/3 proteins, forms an icosahedral particle 95, 96. Fusing GFP to a 49-amino-acid C-terminal fragment of VP2 (VP2C) to create GFP-MPyV VLPs resulted in poor solubility of the anchor protein due to the hydrophobic nature of VP2 97. To improve solubility, VP1 and VP2C-EGFP were co-expressed in prokaryotic cells to form soluble complexes, which were then assembled *in vitro* 98. Reducing the VP2 C-terminal fragment to 31 amino acids improved the encapsulation of EGFP and mRuby3 by MPyV VLPs 98. Similar strategies can be applied to other VLPs such as Simian virus 40 (SV40) 99, JC polyomavirus 100 and Human papillomavirus (HPV) 101 using SP-assisted methods for protein packaging. Overall, employing SPs for VLP protein encapsulation is effective, offering advantages like high efficiency and a single product. However, the fusion of SPs with cargo proteins can sometimes impact the function of the proteins.

### 3.8 Covalent targeted encapsulation

Directly fusing protein genes with CP genes is a widely used method for encapsulating proteins within VLPs (Figure 2H). For instance, infectious bursal disease virus (IBDV) VLPs can incorporate EGFP by fusing it to the N-terminus of VP2 and co-expressing it with wild-type VP2 102, 103. Similarly, reoviruses like rotavirus (RV) and bluetongue virus (BTV) also utilize this direct fusion approach for protein encapsulation 104, 105. In other examples, GFP and red fluorescent protein (RFP) fused to the N-terminus of CPs from canine parvovirus (CPV) and grapevine fanleaf virus (GFLV), respectively, create GFP-CPV and RFP-CPV VLPs 106, 107. While this method effectively integrates specific proteins, its efficiency is constrained by the size of the protein cargo and the assembly of the CPs.

### 3.9 SrtA-mediated encapsulation

Sortase A (SrtA) is another effective tool for encapsulating proteins within VLPs. SrtA selectively cleaves the peptide bond between Thr and Gly residues in the LPXTG signal sequence and then catalyzes the formation of a covalent bond between the C-terminal Thr of the protein and an N-terminal Gly of an oligoglycine peptide 108. This technique has been adapted for VLPs by tagging proteins with the LPXTG signal sequence and covalently binding them to CPs modified with oligoglycines (Figure 2I). For example, in CCMV VLPs, glycine residues are displayed on the CPs' N-terminus inside the particle. When GFP tagged with LPETG is incubated with SrtA, the enzyme facilitates the bonding between GFP and CPs at pH 7.5. Lowering the pH to 5 then promotes VLP assembly and GFP encapsulation, resulting in about 18 GFP molecules per CCMV VLP 109. This approach was also utilized to encapsulate CalB within CCMV VLPs, resulting in an average of 2 CalB molecules per particle 110. The SrtA-mediated approach is advantageous as it requires only short peptide tags, which minimally impact the capsid structure or protein cargo, making it suitable for *in vivo* applications as well. Table 1 provides an overview of various VLP encapsulation methods and their characteristics.

## 4. Application of VLPs encapsulating proteins

VLPs are versatile tools for protein delivery, capable of encapsulating proteins within their core or displaying them on their surface. Their interior cavities can house a diverse array of proteins, including FPs, enzymes, and protein antigens 45. Encapsulated FPs in VLPs are useful for studying VLP packaging mechanisms 85, 111, for intracellular delivery 104, 112, 113, and for *in vivo* imaging 50, 51. Enzymes within VLPs can create biocatalytic nanoreactors for both *in vitro* and *in vivo* applications, offering insights into enzyme function in restricted environments 61, 114, 115. Protein antigens encased in VLPs are effective for developing vaccines against various antigens. On the VLPs' outer surface, proteins can be immobilized or displayed for applications such as enzyme immobilization or vaccine development. Enzyme-VLP complexes can be used to create biosensors or nanoreactors for producing various products. This section highlights the wide-ranging applications of VLPs in protein encapsulation, including their roles in imaging, enzyme therapy, vaccines, immunotherapy, nanoreactors, and biosensors.

### 4.1 Imaging

**Figure 3:**
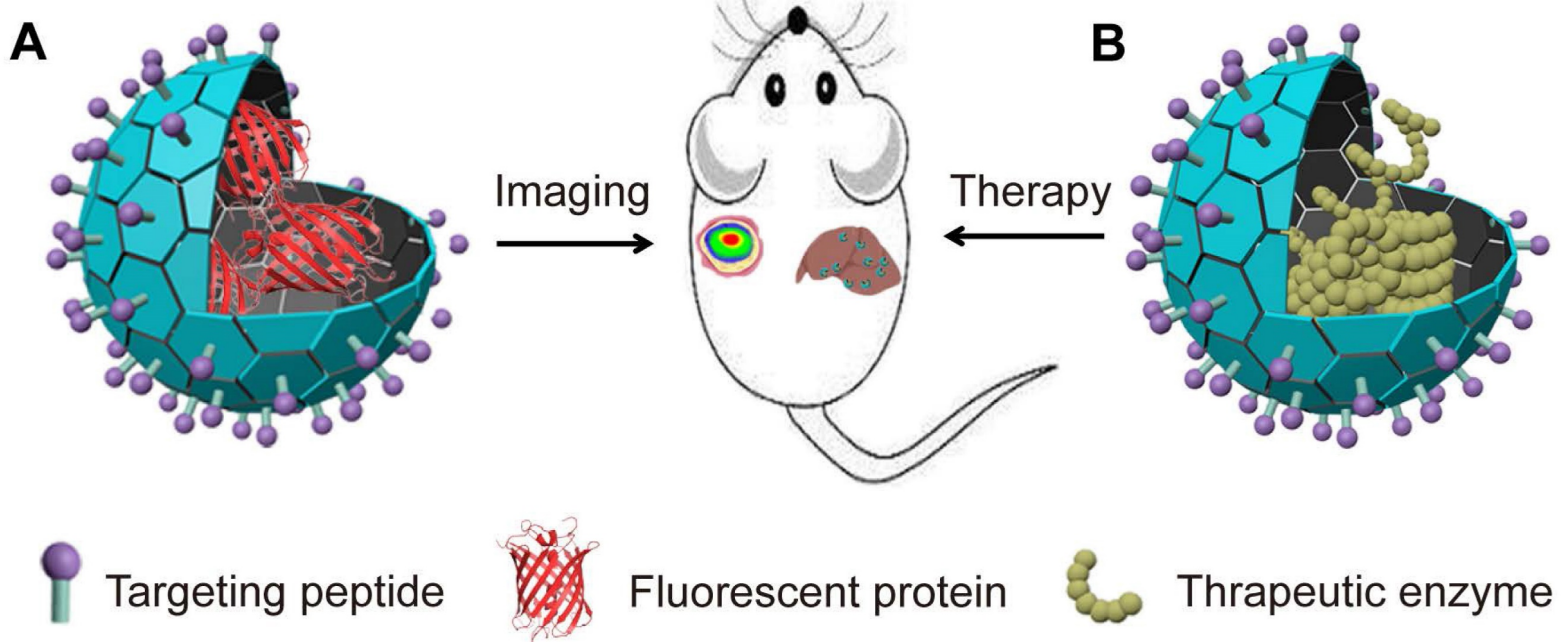
VLPs encapsulating proteins for imaging and ERT. (A). Illustration of VLPs encapsulation FPs for imaging; (B). Illustration of VLPs encapsulation enzyme for ERT.

VLPs can be engineered to encapsulate FPs, creating fluorescent VLPs that are valuable for imaging applications (Figure 3A). These fluorescent VLPs are used for optical imaging, allowing for the specific labeling of cells and tissues, and for studying biological distributions, pharmacokinetics, and interactions 116, 117. By fusing GFP or mCherry to the N-terminus of CPs, fluorescent PVX can be created by controlling the ratio of wild-type CP to FP fusion CP at a 3:1 ratio 112. These fluorescent PVX VLPs can be used to track the viral infection process in plants 118. Similarly, FP iLOV can be fused to the N-terminus of the CPs of PVX, resulting in PVX-iLOV particles. These particles emit a strong fluorescent signal due to the dense arrangement of iLOV FPs, providing a highly sensitive method for imaging 119. Additionally, fluorescent or photoconvertible VLPs can be produced by tagging SARS-CoV-2 proteins, such as the N or M proteins, with fluorescent proteins and mixing these tagged proteins with unlabeled viral components. This technique allows for the study and visualization of the SARS-CoV-2 viral life cycle in a controlled, safe environment 120. The fluorescent SARS-CoV-2 VLPs facilitate imaging of the virus's pathogenic mechanisms *in vivo*.

NIR-FPs are becoming increasingly popular for non-invasive imaging of deep tissues and whole bodies. Their benefits include reduced autofluorescence, minimized light scattering, and lower risk of optical damage. When encapsulated within VLPs, NIR-FPs can achieve greater stability and more targeted delivery to specific cells and tissues due to potential modifications of the VLPs. To assess the imaging performance of these encapsulated NIR-FPs in living organisms, researchers have used Qβ VLPs to package both monomeric mIFP and red-shifted dimeric iRFP720 variants 50. These VLPs retain the photochemical properties of uncoated NIR-FPs but offer improved stability against denaturation and protein degradation. Systemic administration of these VLPs allows for effective visualization of their distribution in the liver 50. Similarly, smURFP encapsulated in Qβ and PP7 VLPs shows distinct tissue and organ localization compared to free smURFP and remains detectable for longer periods 51. Additionally, GFP-Qβ VLPs, which have been modified with ligands for the CD22 receptor, exhibit strong selective binding to CD22+ cells through internalization 67. These results underscore the promising potential of using VLPs to encapsulate NIR-FPs for enhanced diagnostic imaging.

Certain VLPs have also been modified to encapsulate FPs within their interior and display them on their outer surface, highlighting their multifunctionality as nanoplatforms. For example, HK97 VLPs can encapsulate GFPs by fusing GFP to a segment of the GP4 protease 121. Moreover, when the C-terminus of GP5 is tagged with LPETG and incubated with polyglycine-GFP, the enzyme SrtA catalyzes the binding of GFP to GP5. This results in the formation of GFP-HK97 VLPs, which also display GFP on their exterior. These findings establish HK97 VLPs as a versatile nanoparticle platform that can be modularly modified both internally and externally for imaging applications.

### 4.2 Therapeutic protein delivery

VLPs offer a promising method for delivering therapeutic proteins and enzymes. Many diseases arise from enzyme deficiencies or reduced enzyme activity, which disrupt metabolic processes 122. Enzyme replacement therapy (ERT) has become a key treatment for these metabolic disorders 123, 124. ERT involves administering external enzymes to replace the deficient ones within the body 125. However, directly injecting these enzymes can lead to severe immune reactions or degradation by the body's own proteases, which can limit the effectiveness of the treatment. VLPs provide an advantageous solution for these challenges. Their size, biocompatibility, and customizable properties make them ideal for delivering therapeutic enzymes directly to specific targets 126. VLPs enhance the delivery of these enzymes by improving pharmacokinetics, ensuring precise targeting, and increasing overall drug efficacy (Figure 3B) 127, 128. This approach not only optimizes treatment outcomes but also minimizes potential side effects associated with traditional enzyme replacement therapies.

VLPs are emerging as effective carriers for treating metabolic disorders. CCMV VLPs have been used to covalently encapsulate glucocerebrosidase and α-galactosidase A through a specific coupling process 129. To improve delivery, these VLPs were modified with PEG and mannose, enhancing their ability to target lysosomes and effectively treat Gaucher's disease and Fabry's disease 129. Similarly, Brome Mosaic Virus (BMV) VLPs have been used to encapsulate the GALT enzyme, creating GALT-BMV VLPs 130. Mannose-modified BMV VLPs carrying β-glucocerebrosidase have also been developed for Gaucher's disease, demonstrating improved enzyme stability and activity 131. This modification increases the enzyme's stability and extends its half-life in the bloodstream, reducing the frequency of injections and overall treatment costs. VLPs, therefore, represent a major advancement in enhancing the stability, precision, and effectiveness of enzyme replacement therapies.

VLPs have shown significant promise as carriers for therapeutic proteins, offering solutions to some of the challenges faced with traditional cancer treatments, such as drug resistance and severe side effects 132. Many conventional chemotherapy drugs are prodrugs, meaning they need to be activated by specific enzymes to become effective against tumor cells 133. This has led to the development of enzyme prodrug therapy (EPT), a technique that uses targeted enzyme delivery to enhance cancer treatment outcomes 134. For example, Hepatitis B virus (HBV) VLPs can encapsulate yeast cytosine deaminase (yCD), an enzyme that converts the prodrug 5-fluorocytosine (5-FC) into the toxic compound 5-fluorouracil (5-FU). This encapsulation creates yCD-HBV VLPs, which are taken up efficiently by breast cancer cells and exhibit significant toxicity 71. Similarly, SV40 VLPs with yCD can convert 5-FC to 5-FU, leading to cell death in renal fibroblasts 99.

Another approach involves using CCMV VLPs to deliver cytochrome P450 enzymes (CYPs) that activate chemotherapeutic prodrugs like tamoxifen and resveratrol 47. For instance, CYP was fused to P22 VLPs, forming CYP-P22 VLPs. By modifying these VLPs with folic acid, their ability to convert tamoxifen in cancer cells was enhanced, allowing for effective treatment at lower doses 47. Additionally, transfecting human cervical carcinoma cells with P22 VLPs carrying CYPBM3 cytochromes using liposomes boosted CYPBM3 activity up to tenfold compared to natural levels, with CYPBM3-P22 VLPs demonstrating greater stability and activity than free CYPBM3 135. To further improve tamoxifen therapy, CYP-P22 VLPs have been engineered with a photosensitizer and a targeting moiety for combinatorial therapy. These targeted VLPs increase intracellular CYP activity, generate reactive oxygen species (ROS), and significantly enhance tamoxifen's effectiveness, resulting in more potent tumor cell inhibition 136. Beyond enzyme delivery, VLPs can also be used to deliver therapeutic peptides. For instance, P22 VLPs have been employed to encapsulate and deliver two cytotoxic peptides to breast cancer cells, with controlled release triggered by the overexpression of the protease Cathepsin B in these cells 90. This approach demonstrates that VLPs can be customized to improve cancer chemotherapy through both genetic and chemical modifications, offering enhanced precision and effectiveness in targeting tumors.

**Figure 4:**
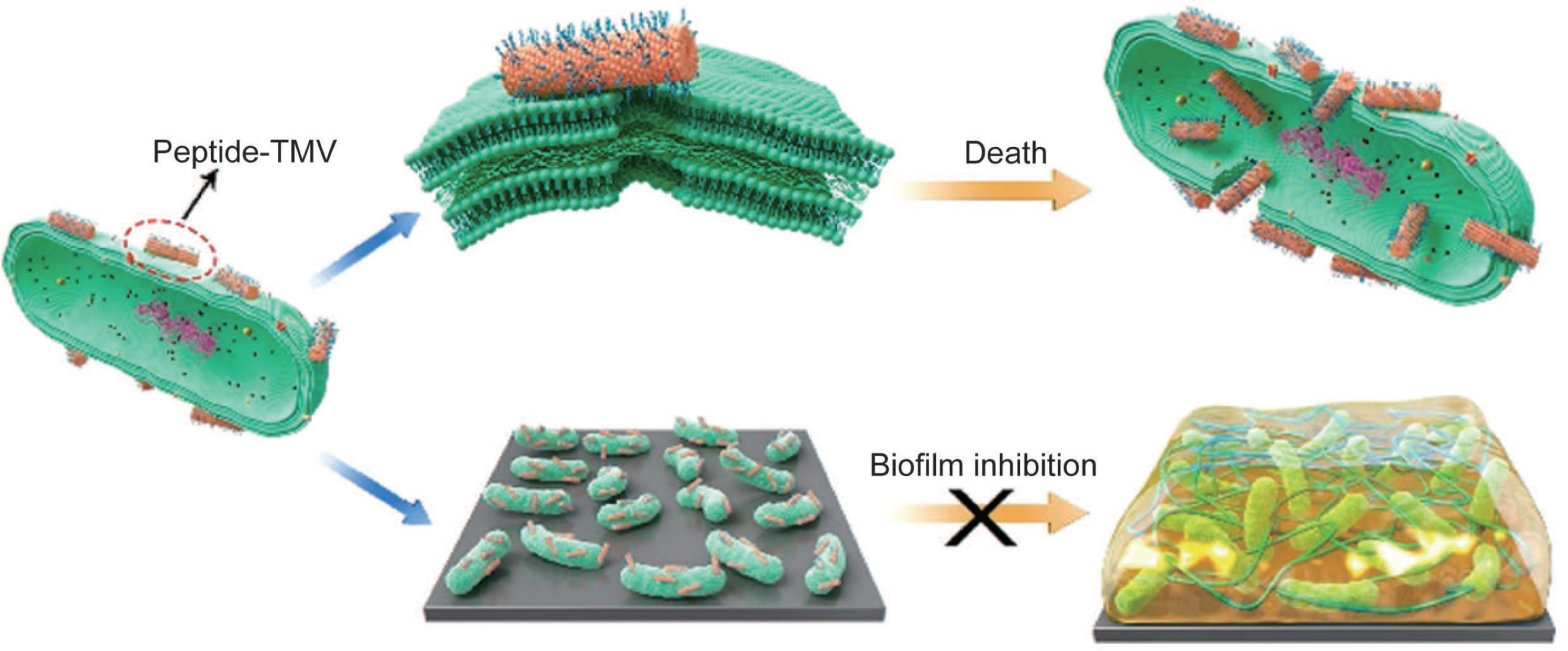
The delivery of AMPs using TMVs could improve the antibacterial efficacy. Reproduced with permission from Ref. 139. Copyright 2021 American Chemical Society.

VLPs have emerged as promising carriers for delivering therapeutic peptides and proteins, particularly in the treatment of bacterial infections. Natural and synthetic antimicrobial peptides (AMPs) are highly effective against bacteria, but their direct use often suffers from issues such as rapid degradation and inadequate targeting 137, 138. Encapsulating AMPs within VLPs addresses these challenges by providing protection and enhancing delivery. For instance, VLPs modified with TAT peptides, such as TAT-TMV VLPs, can effectively target and disrupt bacterial membranes, leading to bacterial cell death 139, 140. These VLPs significantly increase the local concentration of AMPs, demonstrating antibacterial efficacy that is hundreds of times greater than that of free peptides, especially against Gram-negative bacteria (Figure 4) 139. Additionally, T7 phages have been engineered to express a broad-spectrum antimicrobial peptide, 1018, which has shown promise as both an antimicrobial and antibiofilm agent 141. The use of these engineered phages, which deliver AMPs through T7-mediated therapy, further enhances the effectiveness of antibacterial treatments.

In addition to encapsulating peptides and proteins, VLPs can present therapeutic peptides or proteins on their surfaces. For example, M13 VLPs can be modified to display HRP and the Ypep peptide, which targets prostate cancer cells. These VLPs can then catalyze the prodrug indole-3-acetic acid to produce radicals that kill cancer cells 142. Similarly, PVX VLPs can display protein A fragments from *Staphylococcus aureus* and be linked to fluorescent markers for imaging and diagnostic purposes 143. These VLPs have also been engineered to display TRAIL, which effectively targets and treats triple-negative breast cancer in animal models 144. Moreover, VLPs like tissue plasminogen activator (tPA)-TMV, which includes tissue plasminogen activator, have shown improved clinical outcomes by reducing bleeding times compared to free tPA 145. Overall, VLPs used for enzyme or peptide delivery can enhance targeting to tumors, reduce side effects, and improve therapeutic effectiveness.

### 4.3 Vaccine and immunotherapy

**Figure 5:**
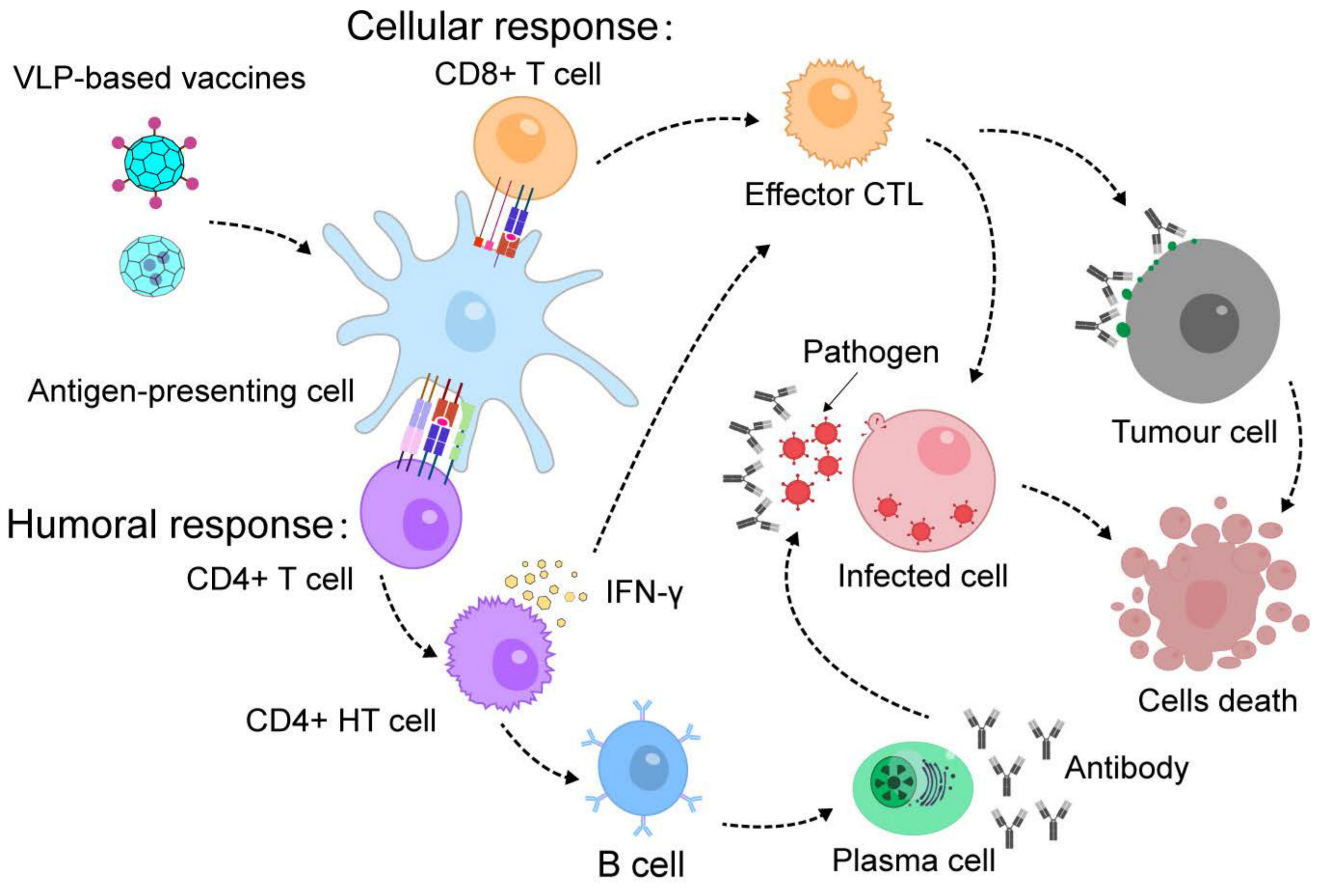
VLP-based vaccines induce immunological memory and confer protection against future infections or cancer immunotherapy.

VLPs are highly effective for developing vaccines and immunotherapies due to their ability to encapsulate or display protein antigens (Figure 5) 146, 147. They also have inherent adjuvant properties that stimulate strong immune responses 148. Chimeric VLPs, which present foreign antigens, have been successful in generating antibodies against pathogens and neutralizing them, thus improving the body's defense against infections 149, 150.

Exogenous antigens can be displayed on the surface of VLPs by attaching them to the ends of their protein components or inserting them into surface loops, which allows for the creation of chimeric VLPs for vaccination 151, 152. For instance, HBc VLPs can be engineered to present enterovirus 71 (EV71) protein and epitopes on their surface through a specific chemical reaction. These chimeric VLPs have been shown to produce effective EV71 antibodies, protecting mice from severe infection 153. Similarly, T4 VLPs can be genetically modified to display antigenic epitopes from both *Bacillus anthracis* and *Yersinia pestis*, leading to a vaccine that offers complete protection against anthrax and plague in animal models 154. Another example involves fusing an antigenic epitope peptides (EPS) from Candida albicans to the side of a phage nanofiber, creating an EPS-displaying phage. Immunization with this phage enhances antibody production and provides protection against *Candida albicans* infection, improving survival rates and reducing fungal loads in mice 155. Additionally, CPMV VLPs and phage VLPs have been used to display SARS and SARS-CoV-2 epitopes, respectively 156-159. These vaccines generate strong immune responses, neutralize viral infections, and offer substantial protection against the viruses. VLPs are also being explored for tumor vaccines, where they display tumor-associated antigens (TAAs) 160-163. Immunization with these VLPs has shown promise in overcoming B-cell tolerance and eliciting strong immune responses, demonstrating their potential as effective platforms for cancer immunotherapy.

VLPs have shown promise not only in fighting infectious diseases but also in stimulating immune responses against chronic conditions. For instance, VLPs derived from murine polyomavirus (MPyV) have been used in cancer immunotherapy. When MPyV VLPs encapsulating human prostate-specific antigen (PSA) were introduced into dendritic cells (DCs), they generated a targeted immune response. This resulted in the production of PSA-specific CD4+ and CD8+ T cells, which effectively protected mice from tumors expressing PSA 164. Similarly, MPyV VLPs incorporating both the extracellular and transmembrane domains of the human Her2 protein have been utilized to prevent the development of Her2-expressing tumors in mice 165. This demonstrates the potential of VLPs to drive therapeutic immune responses against chronic diseases such as cancer.

In addition to presenting proteins on their surfaces, VLPs can also encapsulate proteins within their cores to deliver antigens and boost T cell responses. For instance, P22 VLPs loaded with respiratory syncytial virus (RSV) matrix (M) and matrix 2 (M2) proteins form P22-M/M2 nanoparticles. When administered intranasally to CB6F1/J mice, these nanoparticles prompt antibody production and significantly lower lung viral levels 166. Similarly, IBDV VLPs that encapsulate influenza virus antigens HA2 and M2 produce specific antibodies and reduce mortality in mice during challenge tests 103. Additionally, P22 VLPs encapsulating the influenza nucleoprotein generate a strong T-cell response, providing protection in multistrain challenge assays 167. Similarly, a biomimetic dual-antigen influenza vaccine was constructed by genetically fusing the matrix protein 2 ectodomain (M2e) antigen to the exterior of HBc VLP while encapsulating a conserved nucleoprotein peptide within the VLP. In mice, intraperitoneal immunization with this dual-antigen VLP vaccine elicited specific antibodies against both nucleoprotein and M2e. Furthermore, the biomimetic vaccine group exhibited higher levels of antigen-specific antibodies, more efficient formation of germinal center B cells, and an increased population of effector memory CD8+ T cells 168. Thus, VLPs not only safeguard antigens but also effectively deliver them to immune cells, enhancing humoral immunity by targeting specialized antigen-presenting cells.

### 4.4 Nanoreactor

**Figure 6:**
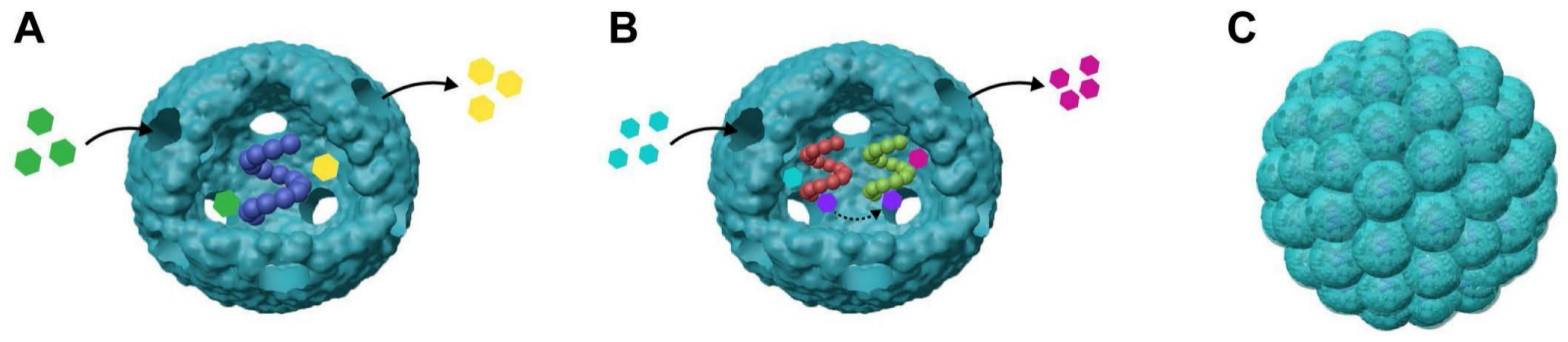
Schematic representation for VLP-based nanoreactors. (A) VLP encapsulating single enzyme for nanoreactors; (B) VLPs encapsulating multiple enzymes to form nanoreactors; (C) VLP-based 3D nanoreactors.

VLPs are used to encapsulate enzymes, creating nanoreactors for biocatalysis and synthetic biology (Figure 6A) 169, 170. Their small pores allow small molecule substrates and products to diffuse in and out, facilitating catalytic reactions without releasing the enzyme. This design improves enzyme stability and bioavailability. Enzymes are often vulnerable to denaturation from heat, chemicals, or proteases, but VLPs can protect them from such degradation. This protective capability has been shown with various VLPs like P22 114, 171, 172, MS2 70, Qβ 68, 123, and CCMV 68, 123. CCMV and P22-based nanoreactors have been used in fundamental studies to explore how confinement affects enzyme activity. VLPs provide structural uniformity and allow precise control over cargo packaging, making them useful for examining how macromolecular crowding impacts enzyme function 87. P22 VLPs were investigated to assess the range of molecule sizes that can diffuse across a porous capsid through the encapsulation of the enzyme AdhD 173. A redox reaction involving PAMAM dendrimer-modified NADH/NAD+ was used to evaluate the size and charge limitations of molecules entering P22. Analysis of the three different morphologies of the P22 particles revealed effective pore sizes, demonstrating that negatively charged substrates diffuse more readily than neutral ones, despite the negatively charged exterior of the particles. For example, research on CCMV VLPs with different amounts of encapsulated lipases showed that enzyme activity was higher at lower encapsulation densities but decreased with higher densities 62. Similarly, studies on P22 VLPs with co-encapsulated ethanol dehydrogenase revealed a negative correlation between enzyme activity and the concentration of structural proteins, indicating that macromolecular crowding affects enzyme dynamics 174.

VLPs can act as nanoreactors for encapsulating single enzymes. For example, P22 VLPs have been used to successfully encapsulate an active [NiFe]-hydrogenase, which is capable of producing hydrogen and tolerating oxygen. This encapsulation not only facilitated the enzyme's inclusion but also stabilized its structure, leading to a nearly 100-fold increase in activity compared to the free enzyme 163. Additionally, this method provided thermal and proteolytic protection, enhancing the enzyme's stability and functionality within the system 171. To further improve enzyme encapsulation, a large negatively charged peptide was added to the C-terminus of *Escherichia coli* alkaline phosphatase (PhoA) before encapsulation into MS2 VLPs. This modification significantly increased the encapsulation efficiency, achieving an average of 1.6 PhoA molecules per VLP 52. This approach not only enhanced the encapsulation process but also ensured that the PhoA enzymes retained their enzymatic activity, demonstrating effective preservation.

VLPs can serve as scaffolds for organizing continuous enzymatic reactions, enabling the creation of biocatalytic cascades (Figure 6B). This method enhances the efficiency of biochemical pathways by reducing intermediate loss from competitive reactions and mimicking the functions of natural catalytic chambers. For example, P22 VLPs have been used to co-encapsulate up to three enzymes in a glycolytic cascade through tandem fusion with self-assembling peptides 84. Similarly, CCMV VLPs, which utilize nucleic acid tags' negative charges, can noncovalently encapsulate various enzymes. Two distinct cascade systems based on glucose oxidase (GOx) were successfully assembled within CCMV VLPs at pH 7.5, demonstrating effective catalytic activity 55. In another approach, MS2 VLPs were employed to co-encapsulate two enzymes involved in indigo biosynthesis through posttranslational fusion. This led to a 60% increase in indigo production in *E. coli*, despite limited control over enzyme loading stoichiometry 70. To further emulate natural compartmentalization, a nested protein cage system was developed using P22 VLPs. In this system, a ferritin protein cage and cellobiose-hydrolase were co-encapsulated through SP fusion, with the ferritin cage acting as a subcompartment within the P22 VLPs 175. These studies highlight the importance of controlling the co-encapsulation of multiple enzymes to effectively organize biocatalytic cascades. Achieving precise *in vivo* organization through defined metabolic pathways in cellular biocatalysis remains a significant challenge in the development of VLP-based biomimetic catalysts.

**Figure 7:**
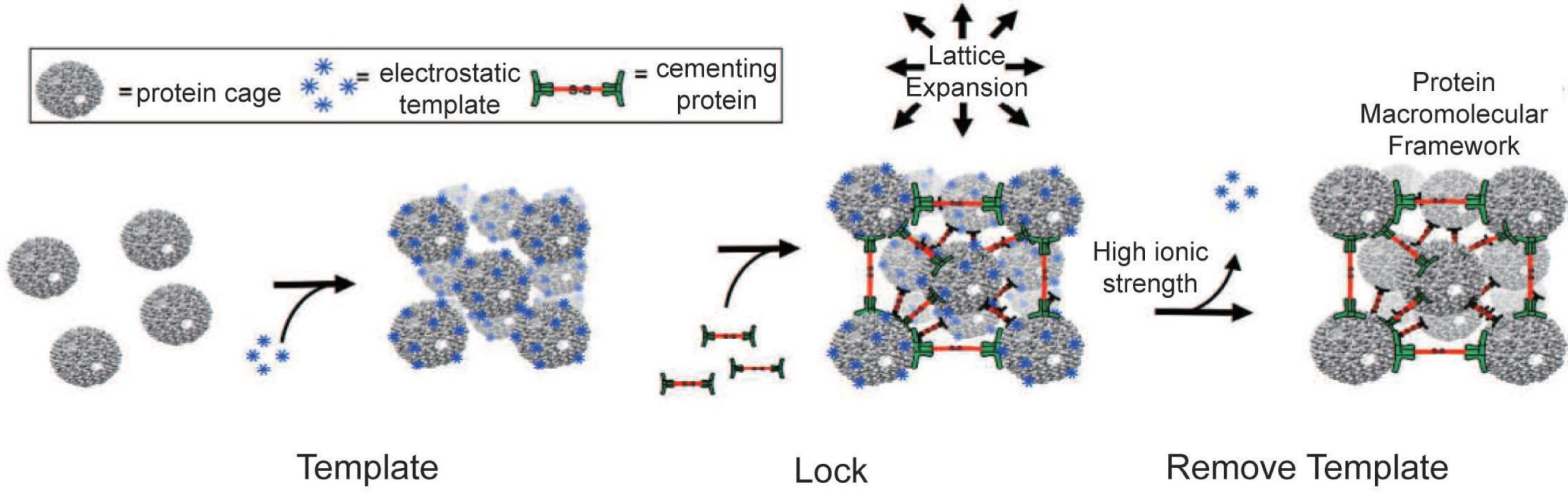
Amine-terminated dendrimers-induced P22 assembly; Reproduced with permission from ref. 181. Copyright 2018 American Chemical Society.

This study explored how VLP-based three-dimensional (3D) protein macromolecular frameworks (PMFs) impact enzyme activity (Figure 6C). VLPs naturally organize into structured arrays, allowing them to form higher-order assemblies like 3D superlattices 176. By combining VLPs with other proteins, ions, or organic compounds, they can be assembled into 3D composites. Examples include CCMV with photosensitive dendrons 177 or avidin 178, P22 VLPs with Dec proteins 179 and DNA-modified Qβ VLPs 180. For instance, using amine-terminated dendrimers as an electrostatic template enables P22 VLPs to encapsulate β-glucosidase, forming a PMF with a high enzyme concentration 181. These dendrimers are stabilized by a binding protein, creating a 3D framework material that enhances catalytic activity (Figure 7) 181. P22 VLPs, when used to encapsulate alcohol dehydrogenase-D enzymes (AdhD), are crucial in developing 3D PMF materials with notable selectivity and activity compared to homogeneous systems 182. Adjusting substrate charge and ionic strength fine-tunes the properties and catalytic rates of these materials. Advanced P22 VLP assembly has improved substrate partitioning, boosting catalytic efficiency for AdhD 182. Additionally, P22 VLPs can encapsulate two different enzymes to form hierarchical 3D arrays using positively charged PAMAM dendrimers 91. These arrays retain enzyme activity and facilitate a coupled two-step reaction, producing isobutanol 91. Such systems also enable efficient recovery of encapsulated enzymes. Overall, VLP-based 3D nanoreactors show significant potential for various applications.

### 4.5 Biosensors

**Figure 8:**
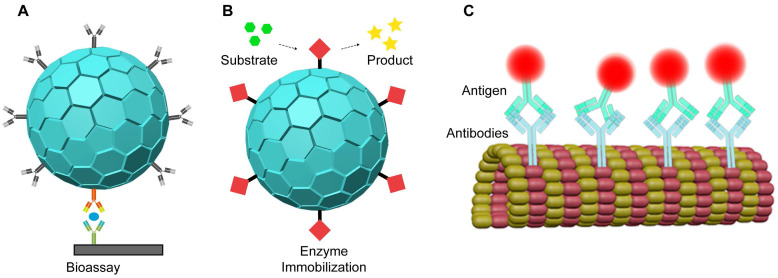
VLPs as proteins immobilization carriers for nanobiosensors.

Biosensors are advanced devices that measure analytes using various signals such as optical, electronic, acoustic, and chemical methods 183, 184. They are highly effective at detecting a wide range of molecules, from small chemicals to harmful biomolecules 185, 186. VLPs have emerged as useful carriers for protein immobilization in optical biosensors (Figure 8A). For instance, enzymes encapsulated in VLPs have been employed in enzyme-linked immunosorbent assays (ELISA) for precise quantification (Figure 8B). In one application, GOx and HRP were immobilized on PEG-biotin modified TMV particles to create a GOX/HRP-TMV biosensor. This biosensor demonstrated enzyme activity 45 times greater than that of free enzymes 79. Similarly, biotinylated TMV nanotubes with glucose oxidase complexes improved glucose detection with high sensitivity, a broad detection range, and rapid response times 80. VLPs are thus valuable for developing sensitive and efficient biosensors.

VLPs can also be used to immobilize or present antibodies for biosensor development (Figure 8C). By genetically engineering Fd phages, specific antibodies can be attached to the pIII protein, while biotin groups on the pVIII protein can bind to avidin-conjugated enzymes 187. Each phage, containing thousands of coat proteins, can carry multiple enzymes, enhancing signal strength by 3 to 4 times in direct ELISA compared to non-phage controls 187. Additionally, Fd phages can be engineered to include magnetic nanoparticles (MNPs)-binding peptides on their surface and epitope peptides for specific interactions with biomarkers, such as the anti-Sap2-IgG for Candida albicans infection 188. These dual-modified phages improve the capture and enrichment of biomarkers in human serum, leading to more accurate quantification of anti-Sap2-IgG using ELISA 188.

**Figure 9:**
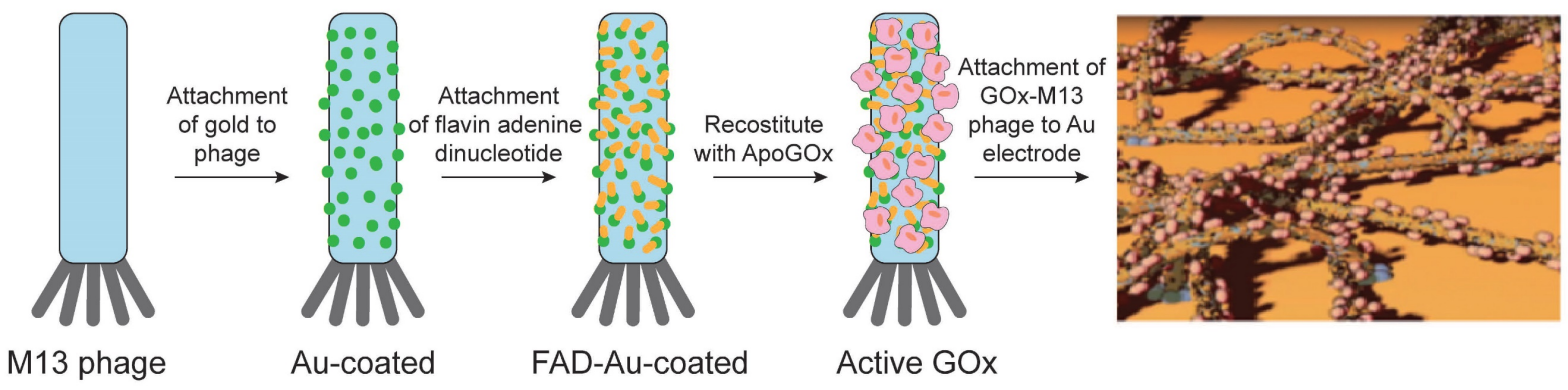
Schematic diagram of assembling the phage/gold/GOx electrode (below). The pIII proteins, shown in red, anchor to a gold substrate, which serves as the electrode. Reproduced with permission from Ref. 192. Copyright 2016 American Chemical Society.

VLPs have been utilized as nanocarriers for enzyme immobilization in the development of electrochemical biosensors, offering several advantages over traditional methods. By using VLPs as enzyme carriers, researchers achieve higher enzyme loading and extended reusability 79, 189. These VLP-based biosensors detect analytes through the substrate specificity of the immobilized enzymes, which bind to target substances and convert them into detectable products. The resulting signals can be read directly through electrochemical devices or through additional reactions involving other enzymes, which produce signals for improved detection. For example, biosensors have been developed by coupling streptavidin-modified penicillinase enzymes to TMV rods using a bifunctional biotin-linker. This approach led to a 1.6-fold increase in enzyme loading and an eight-fold improvement in reusability compared to traditional methods, with a detection limit of 100 μM penicillin G 190. Another application involves biosensor chips that use an array of platinum electrodes loaded with GOx-modified TMV nanotubes for glucose detection. These TMV-based biosensors demonstrate high sensitivity, an extended linear operating range, a low detection limit, and fast response times 191. In a different strategy, M13 VLPs were modified with pIII proteins to attach to a gold substrate, creating a high-surface-area template. GOx was then covalently bound to gold nanoparticles and assembled onto the VLPs using EDC-NHS chemistry. This "nanomesh" structure facilitated direct electron transfer, resulting in a peak current of 1.2 mA/cm² and an enzyme coverage of approximately 4.74 × 10^-8^ mol/cm^2^, which is significantly higher than most existing methods (Figure 9) 192. Overall, these advancements suggest that using VLPs as enzyme carriers in conjunction with electrodes offers a promising approach for creating durable and multifunctional biosensors for on-site health monitoring.

## 5. Conclusion

VLPs offer significant advantages as protein delivery systems. They enhance the stability and activity of proteins, extend their circulation time, improve targeting for treatments or imaging, and reduce side effects by minimizing systemic exposure. To make protein encapsulation into VLPs more efficient and consistent, several methods have been developed. These techniques include random, charge-mediated, coiled-coil-mediated, DNA or RNA aptamer-mediated, SpyTag/SpyCatcher-mediated, scaffolding protein-mediated, covalent targeted, and SrtA-mediated encapsulation. Each method allows VLPs to effectively encapsulate and deliver a range of proteins. Encapsulating functional proteins within VLPs or displaying them on their surfaces has advanced fields such as imaging, enzyme delivery, vaccines, immunotherapy, nanoreactors, and biosensors.

Despite this progress, several challenges still affect the clinical and commercial success of these technologies. One major challenge is ensuring that functional proteins remain active within VLPs. The assembly of VLPs must be precisely controlled to maintain protein stability and functionality. Proper positioning and orientation of proteins are crucial, as spatial constraints within VLPs can impact protein activity. Developing methods to accurately control protein arrangement is essential for achieving effective results. Another issue is maintaining a consistent quantity of protein within each VLP. While this is manageable on a small scale, scaling up to commercial production introduces difficulties in ensuring uniform protein content. This necessitates the optimization of ingredient mixing and process controls to maintain consistency. The internal structure of VLPs also affects protein functionality and stability. Variations in protein distribution can lead to changes in protein conformation, impacting biological activity. Addressing these spatial constraints is key to preserving proper protein folding and function within the VLP. Scaling up from laboratory to commercial production presents additional challenges, such as optimizing fermentation processes, ensuring process stability, and achieving consistency. Separation and purification of VLPs become more complex at larger scales, particularly in distinguishing target VLPs from unencapsulated proteins. Techniques like ultracentrifugation and chromatography need to be finely tuned for high purity and yield.

To optimize VLPs for specific uses, leveraging their natural variability can enhance their physical and chemical properties. Techniques such as site-directed mutagenesis are promising for improving protein loading efficiency, while innovations like high-throughput screening and automated production systems are addressing scalability issues. Directed evolution has proven effective in creating VLPs with diverse forms 193, pH sensitivity 194, and the ability to undergo site-specific modifications 195. Tailoring VLPs with specific surface modifications can enhance targeted delivery and interactions with biological systems, which is a significant step forward in precision medicine. These advancements illustrate how VLPs can be adapted to improve protein encapsulation efficiency for diverse conditions and applications.

Looking ahead, the potential of VLPs for encapsulating and delivering functional proteins is substantial. Future research should focus on understanding how VLPs are taken up by cells, their potential to induce immune responses, and strategies to mitigate these responses. Detailed studies on VLP distribution, clearance, immunogenicity, and toxicity will be vital for realizing their full potential in biomedical applications. Continued research and innovation are essential to fully unlock the capabilities of VLPs in a wide range of applications.

## Figures and Tables

**Table 1:** Methods and characteristics of VLP encapsulating proteins

Encapsulation Method	VLPs Cargo (Molecules per VLP)	Intended Application	Advantage	Disadvantage	Reference
Random Encapsulation	HRP/CCMV, EGFP/HBVc	Targeted delivery, imaging	Straightforward and feasible, without chemical reactions	Lower encapsulation efficiency; requires excess proteins	36, 38
Electrostatic Interaction	1.6 PhoA/MS2, 3 smURFP/Qβ, 1 GOx, 1-2 GCK/CCMV	Enzyme delivery, imaging	Simple and flexible, suitable for charged cargo	Lower encapsulation efficiency, limited loading density	44, 45, 46, 47, 48
Coiled-coil Mediated	15 EGFP/CCMV	theoretical research; nanoreactor	Simple and efficient, high loading density	Genetic modifications may affect protein functionality	40, 55, 56
DNA/RNA Aptamer Mediated	18 PepE, 15 FP, 5-9 NIR-FP/Qβ	Targeted delivery, therapeutic	High efficiency, simple purification steps	Requires aptamer design and optimization	59, 60, 61
SpyTag/ SpyCatcher Mediated	2-4 enzymes/MS2	targeted delivery, imaging	High efficiency, no ligase required	Possible steric constraints on cargo loading density	64, 65, 66
Avidin/Biotin-Mediated	GOx, HRP	Biosensor	Strong, stable binding, versatile	Potential immunogenicity	73, 74
Scaffold Protein Mediated	Aspartase, 249 AdhD, 281 GFP, 233 mCherry/P22	Enzyme encapsulation, therapeutic applications	High loading capacity, versatile methods	Limited control over stoichiometry, potential protein functionality impact	79, 83, 84, 85, 91, 92
Covalent Targeted	EGFP, RFP, CPs	Therapeutic, imaging	High specificity and stability, suitable for chemometrics	Spatial constraints, requires genetic engineering	96, 97, 98, 99,100
SrtA-mediated	18 GFP, 2 CalB/CCMV	Imaging, catalytic studies, therapeutic	Suitable for *in vivo* encapsulation	Requires specific peptide tag	103, 104
